# Visceral obesity in normal-weight patients suffering from chronic schizophrenia

**DOI:** 10.1186/1471-244X-14-35

**Published:** 2014-02-08

**Authors:** Beata Konarzewska, Ewa Stefańska, Agnieszka Wendołowicz, Urszula Cwalina, Anna Golonko, Aleksandra Małus, Urszula Kowzan, Agata Szulc, Leszek Rudzki, Lucyna Ostrowska

**Affiliations:** 1 Department of Psychiatry, Medical University of Bialystok, Plac Brodowicza 1, Choroszcz 16-070, Poland; 2 Department of Dietetics and Clinical Nutrition, Medical University of Bialystok, Mieszka I-go 4B, Białystok 15-054, Poland; 3 Department of Statistics and Medical Informatics, Medical University of Bialystok, Szpitalna 37, Białystok 15-295, Poland; 4 Department of Psychiatry, Medical University of Warsaw, Faculty of Health Sciences, ul. Partyzantow 2/4, Pruszkow 05-802, Poland

**Keywords:** Schizophrenia, Normal weight, Body composition, Bioelectrical impedance

## Abstract

**Background:**

BMI (body mass index) can be misleading regarding the level of adiposity in a normal-weight individual. Recently, a bioelectrical impedance analysis (BIA) method was developed that can measure body composition variables. The main objectives of this study were to use BIA to compare the body composition variables between chronic non-diabetic schizophrenic patients with normal weight and healthy individuals. The secondary objective was to compare the nutritional pattern of schizophrenia patients with that of matched healthy subjects, and to identify possible relationships between the content of different components of their diet and visceral adiposity.

**Methods:**

The subjects were 52 normal-weight patients (33 males and 19 females) diagnosed with schizophrenia based on the DSM-IV and 45 (23 males and 22 females) BMI- matched controls. The patients had been receiving atypical or typical antipsychotic agents for at least one year before enrollment into the study but continuously for 3 months preceding the study and were psychiatrically stable. Body fat (kg), percent (%) body fat, fat-free mass, VAT (visceral adipose tissue) and SAT (subcutaneous adipose tissue) were measured using the bioelectrical impedance analysis (BIA) method. Daily food rations (DFR) were quantitatively evaluated by a 24-h dietary recall method covering 3 days preceding the examination.

**Results:**

In normal-weight patients schizophrenia was significantly linked with higher VAT, VAT/SAT ratio and lower fat- free mass. Men had over 5 times and women over 2 times as much VAT as BMI matched groups. In women with schizophrenia and in their controls, the amount of magnesium, niacin and vitamin B6 in their diet inversely correlated with VAT, while in men lower zinc and vitamin C intake was related to higher visceral adiposity.

**Conclusions:**

Our study has shown that normal-weight patients with chronic schizophrenia have higher levels of visceral fat (VAT) than controls but similar volume of subcutaneous adipose tissue (SAT). Although no clear conclusion can be made regarding cause-and-effect relationships between the dietary content of food served to our patients and visceral obesity, we suggest that schizophrenia diet should be further investigated as a possible factor related to this type of obesity.

## Background

In 1981, Ruderman described the phenotype of “metabolically obese but normal weight” (MONW) individuals [1]. These lean individuals mark a departure from common human patterns, in which a metabolic disease is a consequence of weight gain. MONW subjects display an altered insulin sensitivity and a higher abdominal and visceral adiposity. Visceral obesity puts a strain on the entire circulatory system leading to raised blood cholesterol and triglyceride levels, lower HDL cholesterol levels, increased likelihood of overall cardiovascular risk and associated morbidity and mortality [2,3]. As subjects with visceral obesity might have normal weight, they may escape detection and may not benefit from adequate early prevention of metabolic syndrome [4]. Most schizophrenic patients under neuroleptic treatment gain weight [5,6]. However, some of them maintain a normal weight, despite long-term pharmacotherapy and eating habits similar to those of overweight patients [4]. It is known that patients with schizophrenia make poor dietary choices and they have a tendency to [7,8] have increased caloric intake, including a higher total dietary fat consumption than healthy individuals [8]. Usually, they are also physically inactive; they spend most of the time passively [9-11]. They have higher levels of cortisol and insulin prior to neuroleptic treatment, which make them particularly susceptible to the accumulation of visceral fat [12]. Previous studies which have found an increased visceral adiposity in schizophrenic patients analyzed patients with both normal and abnormal body weight, and not always a test and control group were adjusted for BMI [13,14]. None of them focused entirely on normal-weight patients. These individuals may as well present an unfavorable metabolic body composition and significant visceral obesity even though they had normal body weight.

BMI is a commonly used measure in people with schizophrenia, but there are no data indicating its sensitivity and specificity as an indicator of visceral adiposity among this clinical group. Both BMI and waist circumference are more strongly correlated with subcutaneous (SAT) than with visceral fat (VAT). Although BMI correlates highly with fat mass, depending on level of muscularity, it can be misleading regarding the level of adiposity of an normal weight individual [15]. Recently, a bioelectrical impedance analysis (BIA) method was developed that can measure body composition variables [16]. BIA is based on the principle that there is less resistance to an alternating current passing through tissues that contain fluids and electrolytes than through those containing relatively high amounts of lipids [17]. Its results correlate well with the results of dual energy X-ray absorptiometry (DXA) [18].

The main objectives of this study were to use BIA to compare the body composition variables between chronic non-diabetic schizophrenic patients with normal weight (BMI <25 kg/m^2^) and healthy individuals. We sought to define the magnitude of visceral obesity in both groups in terms of elevated VAT volumes. The second objective was to compare the schizophrenia patients, who in previous studies appeared to have a poor diet [7,8] with matched healthy subjects and to identify possible relationships between the content of different components of their diet and visceral adiposity. We hypothesized that schizophrenia patients food intake which contains inadequate amounts of fibre, vitamins and minerals might be linked with the tendency to accumulate visceral fat.

## Methods

### Participants

This study was conducted between October 2012 and March 2013. The subjects were 52 normal-weight (BMI < 25 kg/m^2^) patients (33 males and 19 females) treated in Psychiatric Hospital, Social Care Unit for Mentally Disabled Patients in Choroszcz and Day Care Center in Bialystok (Poland). They were diagnosed with schizophrenia disorder based on the DSM-IV diagnostic criteria. The age of schizophrenia onset and the length of the disease for males and females were as follows (males 28.9 years; 12.2 years) and (females 25.4 years; 13.2 years). As a reference group, 45 matched healthy subjects (23 males and 22 females) were also included. Patients had been receiving atypical or typical antipsychotic agents for at least one year before enrollment into the study but continuously for 3 months preceding the study and were psychiatrically stable. Medication doses in men and women at the time of the study expressed as chlorpromazine equivalents were as follows (males 891.1 mg; females 722.1 mg).

Sixteen of them have been receiving 1 neuroleptic, 17 patients- 2 or 3 neuroleptics, concomitantly. The most commonly used were: olanzapine, risperidone, haloperidol and clozapine. Exclusion criteria included serious and unstable medical conditions, cognitive disorders, substance dependence within the previous 3 months, known medical conditions which might affect changes in metabolic parameters, known history of diabetes (patients with glucose levels ≥ 126 mg/dL were excluded from the study) or lipid disorder, use of anti-diabetic or lipid-lowering therapy and special diets to lower glucose or lipids levels. The study was approved by the Bioethical Committee of the Medical University of Bialystok, Poland. Informed written consent was obtained from all the subjects after explanation of the nature, purpose, and potential risks of the study.

### Procedures

After providing written informed consent, each subject underwent a physical examination and a psychiatric diagnostic evaluation. Anthropometric measures, including weight, height, umbilicus waist circumference and hip circumference, were obtained. Body composition was measured by means of a bioelectrical impedance analysis (BIA) technique using a Maltron body fat analyzer (Maltron BioScan 920) with an operating frequency of 50 kHz at 800 μA [19]. All measurements were taken around the same time of the day after patients have sat quietly and rested for at least 15 min. The participants lay supine on a non-conducting surface with their arms abducted from the trunk and legs slightly separated for 5 minutes. Four electrodes and cables were attached to the right hand and ankle, as shown in the user’s manual. When the measurements stabilized, the analyzer displayed bioelectrical impedance directly and immediately through the calculation of the software. According to the strong relationships among measured impedance, fat-free mass (FFM), and total body water, many prediction equations were developed to estimate percentage of body fat and FFM [20]. These 2 types of data, the percentage of body fat and FFM, could also be directly displayed after BIA measurement. Previous studies demonstrated excellent test-retest reliability for BIA-obtained measurements, with correlation coefficients ranging from .96 to .99 for resistance measurements [21]. Hydrostatic weighing and BIA-predicted correlation coefficients range from .71 to .93, with standard errors of estimate ranging from 2.7% to 4.7% body fat [22]. The measurement procedure required that the subject should stand barefoot on the analyzer and hold a pair of handgrips, one in each hand. The device uses a multiple-frequency (5 kHz, 50 kHz, 250 kHz, and 500 kHz) BIA technology and has 8 tactile electrodes: 2 are in contact with the palm and thumb of each hand, and 2 are in contact with the anterior and posterior aspect of the sole of each foot. To calculate the body fat, percent (%) body fat, fat- free mass, VAT (visceral adipose tissue), SAT (subcutaneous adipose tissue), muscle mass, and body water for the entire body, the MC-190 uses a proprietary equation developed by the manufacturer.

Daily food rations (DFR) were quantitatively evaluated by a 24-h dietary recall method covering 3 days preceding the examination. The portion sizes of dishes and food products were estimated based on the “Photo Album of Food Products and Dishes” [23]. The energy and nutritional values of the diets were calculated with Diet 5 computer software designed by the Institute of Food and Feeding (IFF) in Warsaw. The findings were compared with the recommended dietary allowance (RDA) or Adequate Intake (AI) proposed by the IFF for healthy adults who report low physical activity [24]. The mean demand for energy and basic nutrients was determined individually referring to due body mass. The intake of protein at 0.9 g/kg of due body mass and fat covering 30% of the anticipated energy demand was referred to as the norm. The carbohydrate demand was calculated from the difference between daily energy demand and the energy derived from protein and fats. The demand for saturated and mono- and polyunsaturated fatty acids was determined at 10%, 12% and 8% of energy demand, respectively. The daily norm for dietary fibre was 30 g, whereas for dietary cholesterol it was not more than 300 mg.

### Statystical analysis

Due to the small size of the groups we used nonparametric tests. To compare the main demographic and clinical characteristics between patients and controls, Mann–Whitney *U* test was performed. Descriptive statistics were calculated as median with the interquartile range: median (IQR). Multiple linear regression analysis was employed to analyze the continuous variables of body composition (independent variables were: gender, the occurrence of schizophrenia, age and BMI). Healthy women were used as the reference category The relationship between selected variables were evaluated using the Spearman correlation coefficient. A value of p < 0.05 was considered significant. The data were analyzed using the package Statistica 10.0 StatSoft.

## Results

The basic characteristics and body composition of participants are presented in Tables 1 and 2.

**Table 1:** Study groups characteristics

	**Male**			**Female**		
	**Schizophrenia N = 33**	**Controls N = 23**	**P value**	**Schizophrenia N = 19**	**Controls N = 22**	**P value**
Age (years)	46 (31, 57)	42 (27, 53)	0.368	41 (30, 47)	39.5 (30, 47)	0.990
Body weight (kg)	70 (68, 75)	74.4 (66, 77)	0.133	59 (54, 68)	58.5 (54.7, 64.2)	0.783
Height (cm)	175 (172, 178)	179 (170, 186)	0.236	164 (161, 170)	167.5 (163, 170)	0.511
BMI	23.3 (22.3, 24)	23.5 (22.3, 24.2)	0.516	21.8 (20.8, 24.1)	20.9 (20.1, 22.1)	0.302
WHR	0.9 (0.9, 0.9)	0.9 (0.9, 0.9)	0.434	0.89 (0.85, 0.92)	0.84 (0.79, 0.86)	0.009

**Table 2:** Body composition measurements of patients with schizophrenia and controls

	**Males**			**Females**		
	**Schizophrenia N = 33**	**Controls N = 23**	**P value**	**Schizophrenia N = 19**	**Controls N = 21**	**P value**
Fat free mass (kg)	57.2 (54, 59.9)	62.5 (54.8, 66)	0.010	43.4 (41.8, 45.6)	44.7 (42.6, 46.1)	0.229
Fat free mass (%)	81.2 (78.8, 85)	83.9 (78.5, 86.5)	0.286	72.8 (70.1, 77.4)	74.5 (70.1, 77.3)	0.804
Body fat (kg)	13.3 (10, 15.7)	11.5 (10.3, 16.3)	0.653	16 (12.2, 19.3)	15.3 (12.5, 18.3)	0.824
% Body fat	18.8 (14.4, 21.2)	16.1 (13.5, 19.7)	0.194	27.2 (22.6, 29.9)	25.5 (22.7, 29.9)	0.695
RMR (kcal)	1798 (1585, 1885)	1820 (1704, 2120)	0.050	1427 (1390, 1517)	1479 (1435, 1520)	0.472
VAT (cm^2^)	351.9 (262, 586.8)	73 (52, 201)	0.000	124 (56, 281)	55.5 (39, 84)	0.006
SAT (cm^2^)	88 (80, 112)	108 (78, 139)	0.286	82 (65, 92)	80.5 (73, 101)	0.565
VAT/SAT ratio	3.5 (2.5, 5)	0.7 (0.5, 1.9)	0.000	1.4 (0.6, 3.5)	0.7 (0.5, 1)	0.005

There were no statistical between-group differences in age, body mass, height and BMI. In females, WHR (waist hip ratio) was significantly higher in patients with schizophrenia than in healthy individuals. RMR (resting metabolic rate) and fat free mass (kg), were in all groups positively correlated (males with schizophrenia r = 073, p = <0.001, n = 33, controls r = 0.83, p = p = <0.001, n = 23; women schizophrenia 0.53, p = 0.02, n = 19, controls r = 0.56, p = 0.007, n = 22). Furthermore, RMR and fat free mass were significantly lower in patients with schizophrenia than in healthy individuals (Table 1). Both groups of men and women did not differ with regard to the SAT. In both males and females VAT and VAT/SAT ratio were significantly higher in patients with schizophrenia than in healthy individuals (Table 2). Within schizophrenia groups the only demographic variable that correlated positively with visceral obesity, namely with VAT/SAT ratio was the length of the disease, however, this type of relationship was observed exclusively among males (Spearman correlation r = 0.51, p = 0.01, n = 23). There were no significant differences between men and women with respect to the onset of schizophrenia and the duration of the disease.

Multiple regression analysis for the detailed body composition characteristics. Healthy women were used as the reference category.

The results of the regression analyses examining the associations between the diagnosis of schizophrenia and body composition in normal-weight patients with schizophrenia are presented in Table 3. Schizophrenia was significantly associated with lower fat- free mass (kg), higher VAT and VAT/SAT ratio. There was a close relationship between the male gender and higher VAT and VAT/SAT ratio. Subjects’ age and BMI were positively related to higher values of fat mass (kg) but inversely with % fat free mass.

**Table 3:** Multiple regression analysis for the detailed body composition characteristic

		**Beta coefficient**	**P value**	**R**^**2**^
Fat free mass (kg)				
	Gender	14.101	P < 0.001	
	Schizophrenia	-2.990	0.001	0.757
	Age	-0.134	P < 0.001	
	BMI	0.925	P = 0.001	
Fat free mass (%)				
	Gender	11.546	P < 0.001	
	Schizophrenia	-0.496	0.376	0.801
	Age	-0.105	P < 0.001	
	BMI	-2.123	P < 0.001	
Body fat (kg)				
	Gender	-6.109	P < 0.001	
	Schizophrenia	-0.063	0.899	0.697
	Age	0.006	0.004	
	BMI	2.023	P < 0.001	
Body fat (%)				
	Gender	-11.642	P < 0.001	
	Schizoprenia	0.646	0.268	0.788
	Age	0.106	P < 0.001	
	BMI	2.051	P < 0.001	
VAT				
	Gender	169.451	<0.001	
	Schizophrenia	201.670	P < 0.001	0.325
	Age	1.170	0.457	
	BMI	-4.729	0.701	
VAT/SAT				
	Gender	1.585	0.001	
	Schizophrenia	2.098	P < 0.001	0.280
	Age	0.019	0.265	
	BMI	-0.171	0.207	

The analysis of the food intake showed that daily food rations of men with schizophrenia compared to those of healthy subjects provided less kcal/day. Also glucose, proteins and fibre consumption was lower in this group. On the other hand, the mean intake of saturated fats in the diets of women with schizophrenia was higher compared to controls (Tables 4 and 5). Daily food rations of men with schizophrenia provided lower amounts of vitamins (B2, C) and minerals (zinc, magnesium, iron, copper, calcium). Additionally, the intake of vitamin D3, C, folic acid, calcium, magnesium in men, and in women with schizophrenia also iron, did not cover daily requirements for these components (RDA) (Tables 6 and 7).

**Table 4:** Energy and nutritional value of daily food rations of the male study participants

	**Schizophrenia (N = 27)**		**Controls (N = 22)**		
	**Median (IQR)**	**% realization of the norm and recommendations**	**Median (IQR)**	**% realization of the norm and recommendations**	**P value**
Energy (kcal)	1719.9 (1464.8, 2313.3)	63.7	2114.4 (1855.5, 2722)	78.3	0.021
Water (ml)	1537 (1234.4, 1820.6)	41.5	1700.4 (1413.8, 2462.4)	45.9	0.07
Proteins (total) (g)	62.6 (52.8, 86)	77.2	102.2 (68.9, 108.5)	126.1	0.004
Proteins (animal) (g)	44.1 (32.9, 52.2)	110.2	70.2 (40.9, 85.2)	175.5	0.015
Fat (g)	67.2 (48, 91.3)	74.6	73.4 (53.7, 103.2)	81.5	0.594
Carbohydrates total (g)	235.2 (152.6, 274.4)	60.1	320.3 (219.3, 374.7)	81.9	0.011
Saturated fatty acids (g)	32.2 (17.8, 39.7)	107.3	27.9 (22.1, 39.6)	93.0	0.786
Monounsaturated fatty acids (g)	27.4 (19.5, 39.6)	76.1	27.5 (21, 150.3)	76.3	0.771
Polyunsaturated fatty acids (g)	6.8 (4.1, 9.8)	28.3	7.9 (5.7, 12.6)	32.9	0.140
Cholesterol (mg)	240.7 (187.3, 417)	80.2	306.2 (233.1, 484.6)	102.0	0.540
Fibre (mg)	14.8 (49.3%) (11.4, 18.1)	49.3	18.3 (14.5, 25.7)	61.0	0.041

**Table 5:** Energy and nutritional value of daily food rations of the female participants

	**Schizophrenia (N = 17)**		**Controls (N = 20)**		
	**Median (IQR)**	**% realization of the norm and recommendation**	**Median (IQR)**	**% realization of the norm and recommendation**	**P value**
Energy (kcal)	1621.3 (1460.1, 1915.3)	77.2	1378.8 (1131, 1636)	65.6	0.057
Water (ml)	1561.4 (1376.4, 1711.3)	57.8	1804.5 (1386.3, 2230.8)	66.8	0.156
Proteins (total) (g)	64.2 (53.8, 69.1)	101.9	59.5 (54.3, 93.1)	94.4	0.532
Proteins (animal) (g)	37.8 (29.7, 48.5)	118.1	45.9 (35.9, 67.8)	143.4	0.065
Fat (g)	44.3 (40.1, 58.5)	63.3	37.8 (25.9, 57.5)	54	0.097
Carbohydrates total (g)	253.3 (183, 306.5)	83.3	182.8 (144.9, 221.2)	60.1	0.070
Saturated fatty acids (g)	20 (16.5, 27.9)	86.9	14.4 (10.4, 20.1)	62.6	0.034
Monounsaturated fatty acids (g)	17.4 (16.3, 29.2)	62.1	14.4 (9.7, 23)	51.4	0.110
Polyunsaturated fatty acids (g)	5.2 (4.2, 5.6)	27.4	4.4 (3.7, 5.5)	23.1	0.353
Cholesterol (mg)	190.7 (157.6, 252.4)	63.6	194.8 (127.8, 266.5)	64.9	0.915
Fibre (mg)	16.2 (13.3, 209)	54	14.9 (12.1, 17.4)	49.6	0.511

**Table 6:** Supply of vitamins and minerals in daily food rations (male participants)

	**Schizophrenia (N = 27)**		**Controls (N = 22)**		
	**Median (IQR)**	**% realization of the norm and recommendations**	**Median (IQR)**	**% realization of the norm and recommendations**	**P value**
Ca (mg)	390.4 (247.8, 588.3)	39.0 (AI)	580.6 (353.4, 940.3)	58.1 (AI)	0.029
P (mg)	896.9 (797, 1172.4)	128.1 (RDA)	1411.2 (1019.2, 1868.7)	201.6 (RDA)	0.002
Mg (mg)	227.1 (165.7, 293)	54.1 (RDA)	314.4 (221.5, 410.4)	74.8 (RDA)	0.003
Fe (mg)	8.5 (7.3, 11.4)	85.0 (RDA)	12.4 (8.6, 14.1)	124.0 (RDA)	0.025
Zn (mg)	8.4 (7.7, 11 8)	76.4 (RDA)	11.2 (8.8, 15.7)	101.8 (RDA)	0.031
Cu (mg)	0.9 (0.7, 1.1)	100 (RDA)	1.3 (0.9, 1 5)	144.4 (RDA)	0.003
B1 (mg)	1.3 (0.9, 1.8)	100 (RDA)	1.5 (1.1, 2.8)	115.3 (RDA)	0.101
B2 (mg)	1.2 (0.9, 1.6)	92.3 (RDA)	1.7 (1.3, 2.5)	130.7 (RDA)	0.007
Niacyn (mg)	13.7 (11, 17.8)	85.6 (RDA)	17.7 (11, 30.3)	110.6 (RDA)	0.145
B6 (mg)	1.7 (1.3, 2.1)	130.7 (RDA)	2.1 (1.5, 2.6)	161.5 (RDA)	0.115
C (mg)	29.4 (21.8, 58.7)	32.6 (RDA)	67.6 (31.3, 105.4)	75.1 (RDA)	0.024
Folic acid (μg)	187 (139.8, 235.8)	46.7 (RDA)	242.2 (176.4, 288.2)	60.5 (RDA)	0.075
D (μg)	1.9 (1.3, 3.3)	38 (AI)	2.3 (1.6, 3.6)	46.0 (AI)	0.282

RDA-Recommended Dietary Allowances; AI-Adequate Intake.

**Table 7:** Supply of vitamins and minerals in daily food rations (female participants)

	**Schizophrenia (N = 17)**		**Controls (N = 20)**		
	**Median (IQR)**	**% realization of the norm and recommendations**	**Median (IQR)**	**% realization of the norm and recommendations**	**P value**
Ca (mg)	474.1 (208.6, 616.3)	47.4 (AI)	434.6 (280.2, 612.1)	43.5 (AI)	0.988
P (mg)	1068.6 (806.5, 1262.1)	152.6 (RDA)	1088 (837.5, 1248.7)	108.8 (RDA)	0.796
Mg (mg)	261.7 (175.2, 360.3)	81.8 (RDA)	222.8 (177.5, 306.1)	69.6 (RDA)	0.939
Fe (mg)	8.4 (7.4, 10)	46.6 (RDA)	7.4 (6.1, 10.6)	41.1 (RDA)	0.512
Zn (mg)	8.6 (7.3, 9.9)	107.5 (RDA)	8.2 (5.5, 10.4)	102.5 (RDA)	0.552
Cu (mg)	0.8 (0.6, 1.1)	88.8 (RDA)	0.9 (0.7, 1.1)	100 (RDA)	0.784
B1 (mg)	1.0 (0.8, 1.2)	90.9 (RDA)	1.1 (0.8, 1.3)	90.9 (RDA)	0.879
B2 (mg)	1.2 (0.8, 1.5)	109 (RDA)	1.3 (1.2, 1.6)	108.3 (RDA)	0.377
Niacyn (mg)	10.3 (6.4,16.7)	73.6 (RDA)	12.7 (8.3, 24.3)	90.7 (RDA)	0.124
B6 (mg)	1.3 (1, 1.5)	100 (RDA)	1.5 (1.4, 1.7)	115.4 (RDA)	0.097
C (mg)	31.9 (17.5, 40.7)	42.5 (RDA)	60.7 (32.6, 108.9)	80.9 (RDA)	0.020
Folic acid (μg)	164.7 (139.8, 204.9)	41.2 (RDA)	186.6 (161, 231.8)	46.6 (RDA)	0.110
D (μg)	1.3 (0.9, 2.1)	26 (AI)	1.6 (0.9, 2.1)	32 (AI)	0.927

RDA-Recommended Dietary Allowances; AI-Adequate Intake.

In female group, the presence of high amount of saturated fatty acids in their diet was positively related to fat mass (kg) (women with schizophrenia, r = 0.73, p = 0.0007, n = 17). In women with schizophrenia and in their controls, the amount of magnesium, niacin, vitamin B6, and in men diagnosed with schizophrenia, lower zinc and vitamin C, was inversely correlated with the VAT or VAT/SAT ratio (Table 8).

**Table 8:** **Spearman rank correlations between nutrients and VAT**^**1 **^**or VAT/SAT ratio**^**2**^

	**Female**					**Males**			
	**Schizophrenia**		**Controls**			**Schizophrenia**		**Controls**	
	**R**	**P**	**R**	**P**		**R**	**P**	**R**	**P**
Niacin	-0.50	0.04^2^	-0.52	0.02^2^	Vit. C	-0.47	0.01^2^	-0.51	0.01^2^
Vit. B6	-0.63	0.006^1^	-0.5	0.02^2^	Zn	-0.45	0.02^2^	-	-
Mg	-0.60	0.01^1^	-0.47	0.04^1^	-	-	-	-	-

## Discussion

This study was the first to compare body composition of normal-weight, non-diabetic, chronic schizophrenia patients with that of healthy controls. The main findings of this study were that both normal-weight men and women with schizophrenia had visceral fat content significantly higher compared to that of the control groups, while there was no difference with respect to the amount of subcutaneous adipose tissue (SAT). Although in both males and females with schizophrenia visceral adiposity was higher compared to controls, mainly the male gender was associated with higher values of VAT, VAT/SAT ratio. The mechanism for increased visceral fat in schizophrenic patients has not been completely elucidated. Patients with schizophrenia are at risk of developing obesity due to poor dietary habits, lower resting energy expenditure, lack of exercise or limited activity due to negative symptoms of schizophrenia. Previous studies have suggested an increased propensity for storing excess fat as visceral adiposity in schizophrenic patients [12,14]. Ryan at al. (2004) have shown that even first episode, drug-naïve patients with schizophrenia had over three times as much visceral fat as BMI-matched controls. In their study, drug-naïve patients with schizophrenia were hypercortisolemic, consumed less fibre though more saturated fat, had higher WHRs, and had just over 3 times as much VAT as the BMI matched group of control subjects [12]. According to the authors, the most likely pathophysiological mechanism underlying this increase in visceral obesity in schizophrenia was hypercortisolemia. High levels of cortisol primarily increase LPL activity, and do so to a greater extent in VAT fat cells, thereby leading to a greater deposition of visceral fat [25]. Ryan et al. (2004) also analysed the impact of risperidone and olanzapine treatment on VAT and concluded that they do not appear to play a part in the generation of excessive levels of visceral fat in their group of never-treated patients with schizophrenia [12].

Our sample, in contrast to the previous study, consisted of patients with normal weight (BMI < 25 kg/m^2^) for many years exposed to neuroleptic treatment. Males were more prone to higher VAT levels although the mean intake of absorbable carbohydrates and saturated fats in the diets of women with schizophrenia was higher, as compared to controls. Men had over 5 times, while women over 2 times as much VAT as the BMI-matched groups did. Both BMI and age of the patients did not affect the contents of the VAT. Higher VAT and lower fat free mass (kg) was directly related to schizophrenia, while body fat mass positively correlated with the subjects’ age and BMI. Our results turned to be only partly consistent with other studies investigating body mass composition in schizophrenia patients chronically exposed to neuroleptic treatment. In a study by Sugawara et al. (2012), schizophrenia was significantly linked with more body fat, higher % body fat and lower fat- free mass [26]. Contrary to men, in women schizophrenia had a significant association with lower % body fat, higher fat- free mass, higher muscle mass. In turn, Nilsson et al. (2006) found that patients with schizophrenia had higher % body fat and lower fat- free mass than healthy controls [27]. Saarni et al. (2009) reported that schizophrenia was significantly associated with a higher % body fat and lower fat- free mass after adjusting for age, gender, and BMI [13]. Nevertheless, all of these studies showed smaller content of the fat free mass in patients with schizophrenia. The differences between our results and data from other studies may be due to the fact that the previous studies included patients who were overweight, samples were not always BMI- matched and the body fat content was not differentiated in terms of its location: into visceral and subcutaneous fat depot. Most previous studies also used different methods than BIA, and analyzed a mixed subject pool of both genders or only male subjects.

In our chronically treated patients, the only demographic variable that correlated positively with visceral depot besides schizophrenia was the length of the disease. However, this type of relationship was observed exclusively among males. This may be partially explained by the use of antipsychotic medication and unhealthy lifestyle habits. Antipsychotic medications, especially atypical, have been associated with significant weight gain and visceral obesity [28]. In Zhang et al. (2004) study an antipsychotic drug treatment rapidly induces abdominal fat deposition and hyperlipidaemia. Therefore, it may be possible for older subjects with a longer duration of the disease to have higher VAT, since normally they have been on antipsychotic medication for a longer period of time.

Another factor which was related with the amount of VAT in our study was the components of the patients’ diet. In women with schizophrenia, the presence of high amount of saturated fatty acids in their diet was positively related to fat mass. The analysis of the food intake showed that daily food rations of men diagnosed with schizophrenia provided less calories, proteins, glucose and fibre compared to controls, whereas women, while providing a similar amount of calories, tend to eat more saturated fatty acids. The results of our study point to the fact that patients with schizophrenia with normal weight do not have a tendency to consume excessive amounts of calories, quite the contrary. Thus, the accumulation of visceral fat in this group may be due to improper composition of the food rather than due to excessive daily calorie intake. Daily food rations of men diagnosed with schizophrenia provided also smaller amounts of vitamins (B2, C) and minerals (zinc, magnesium, iron, copper, calcium). In women with schizophrenia and in their controls, the amount of magnesium, niacin and vitamin B6 in their diet was inversely correlated with the VAT, while in men lower zinc and vitamin C intake was related to higher visceral adiposity. Numerous studies have provided evidence that the above-mentioned components of the diet are associated with energy metabolism. Magnesium has been linked with a number of chronic diseases, including diabetes, hypertension and lipid abnormalities [29]. Magnesium deficiency [30] was described a common factor associated with insulin resistance [31], which impairs energy metabolism efficiency and reduces the capacity for physical work, exerts negative effects on blood glucose homeostasis, and is independently associated with depressive symptoms [32-35]. In addition, magnesium intake in two studies was inversely associated with the metabolic syndrome and risk of type 2 DM [36]. In turn, niacin has been reported to decrease VLDL, LDL and small dense LDL particles and increase HDL levels resulting in an overall beneficial lipoprotein profile. Niacin in Kamanna and Kashyap (2000) study reduced plasma free fatty acid levels by inhibiting adipose tissue lipolysis, thereby decreasing available substrate for hepatic VLDL synthesis [37]. Niacin, alone or in combination with statins, has demonstrated effectiveness in reducing the progression of atherosclerosis in hypertriglyceridemic individuals with coronary disease in old or small studies [37-39]. Vitamin B_6_, although not directly related with energy metabolism, is essential for the brain regional synthesis of 5-HT (serotonin). In turn, serotonin has been implicated in various CNS functions that regulate sleep onset, blood pressure, mood but also food intake satiety [40,41]. In turn, zinc ions in Chen et al. study (1988) influenced adipose tissue metabolism by regulating leptin secretion and by promoting free fatty acid release and glucose uptake [42]. Apart from participating in the control of leptin secretion, zinc also seems to be intimately involved in glucose- and fat-metabolism in adipocytes, where zinc per se has a role as an insulinomimetic [43]. There is clear evidence that visceral obesity is associated with increased oxidative stress and inflammation [44]. Another recent data also suggest that dietary antioxidants, including vitamin C intake may be a predictor of the risk to develop metabolic syndrome features such as adiposity or impairments in systolic blood pressure, serum glucose and free fatty acids, and some inflammatory biomarkers in healthy subjects [45].

## Conclusions

It is difficult to draw conclusions from this study as its limitations include a relatively small sample size and the fact that daily food rations were evaluated by a method covering 3 days preceding the examination. Despite these shortcomings, our study has shown that normal weight patients with chronic schizophrenia have higher levels of visceral fat than controls but a similar volume of SAT. Although in both males and females with schizophrenia the visceral adiposity was higher compared to controls, mainly the male gender was associated with higher values of VAT and the VAT/SAT ratio. Schizophrenia patients have poor nutritional patterns. Although no clear conclusion can be drawn regarding cause-and-effect relationships between the dietary content of food served to our patients and visceral obesity we suggest that schizophrenic diet should be further investigated as a possible factor related to this type of obesity.

## Competing interests

The authors of this manuscript have no conflicts of interest to disclose as described by the Journal.

## Authors’ contributions

BK conceived the study, designed the study, interpreted the data and wrote the initial draft of the manuscript. AS, ES and LO contributed to study design and assisted in drafting the manuscript. UC conducted the statistical analysis. ES, AW, UC had full access to all of the data in the study and take responsibility for the integrity of the data and the accuracy of the data analysis. UK, ES, AW, AG, AM, LO, LR completed recruitment of participants. All authors have approved the manuscript.

## Pre-publication history

The pre-publication history for this paper can be accessed here:

http://www.biomedcentral.com/1471-244X/14/35/prepub
